# Electroconvulsive Therapy in Transgender and Gender Diverse Population: A Case Report and Review of Literature

**DOI:** 10.1155/2024/5552781

**Published:** 2024-05-07

**Authors:** Lachlan Draper, Ashis Vikas, Subhash Das, Suresh Yadav, Frances Walker, Ivona Bandilovska

**Affiliations:** ^1^North West Area Mental Health Service Northern Health, Melbourne, VIC, Australia; ^2^Northern Area Mental Health Service Northern Health, Melbourne, VIC, Australia; ^3^The University of Melbourne, Melbourne, VIC, Australia

## Abstract

**Objective:**

Present a case of a transgender and gender diverse (TGD) individual receiving gender affirming hormone therapy (GAHT) who presented with first episode bipolar mania and received electroconvulsive therapy (ECT). To understand the safety and efficacy of ECT in the TGD population including those receiving GAHT through literature review.

**Materials and Methods:**

Case report using informed consent from an individual TGD patient who received ECT. A review of the literature was conducted using PubMed, Embase, and Medline.

**Results:**

The case illustrated safe and effective ECT use in a TGD individual receiving GAHT. Eight studies were reviewed. GAHT has been reported to interfere with certain anaesthetic agents used in ECT. ECT appeared to be a safe and effective treatment in the TGD samples in those studies.

**Conclusion:**

There is limited literature on the safety and efficacy of ECT for TGD individuals receiving GAHT. More research is required to address mental health inequalities in this population and to support safe and effective gender affirming treatment modalities.

## 1. Introduction

Estimates suggest 0.4%–1.3% of the global population are transgender and gender diverse (TGD) [1]. There is a higher prevalence of mental illness in TGD individuals compared to the general population [2]. Increasing numbers of TGD individuals are presenting to health services for gender affirming hormone therapy (GAHT) [3, 4]. While GAHT is considered safe and has been demonstrated to improve psychosocial functioning [5], it requires monitoring for adverse effects and mood changes during the transition period.

There is a paucity in health data of the TGD global population [6], which is in part due to the challenges in accurately capturing demographic data at both a population and health service level. Therefore, those TGD individuals presenting with severe mental illness are especially vulnerable, given the lack of evidence base of treatment efficacy and unclear risk of adverse effects in their population.

Electroconvulsive therapy (ECT) is an effective treatment for several psychiatric disorders [7]. The authors present a case report of a patient receiving GAHT who presented with the first episode of bipolar mania with life threatening symptoms requiring ECT, and a literature review summarising the current evidence base of ECT use in the TGD population.

## 2. Case Report

### 2.1. Method

Written informed consent was provided by the patient. Patient details have been anonymised and identifiable information has been removed. The case report was registered with the Northern Health Research Development and Governance Unit. No further ethical approval was required.

### 2.2. Case

LJ, a 25-year-old person (preferring they/them pronouns) transitioning from female to male and receiving GAHT, was brought to hospital by ambulance after their partner noticed new erratic and bizarre behaviour. LJ had a past psychiatric history significant for anxiety, major depressive disorder (MDD), and post-traumatic stress disorder (PTSD). They saw a psychologist regularly in the community and had sequentially trialled duloxetine and sertraline, the latter of which was being weaned prior to admission. They had been receiving topical testosterone 1% as GAHT for approximately 12 months prior to presentation with no recent changes in formulation or dose. They drank alcohol and smoked cannabis socially, with no other drug use prior to admission. A family history of bipolar affective disorder (BPAD) in LJ's maternal uncle and grandfather was recorded.

During initial reviews LJ presented with features of psychosis as well as affective lability, giving an initial impression of bipolar mania with psychosis. Oral olanzapine (20 mg daily total) and lithium sustained release (450 mg twice daily) were commenced. In the days following admission, LJ's condition deteriorated with signs of emerging catatonia with deteriorating oral intake and emerging lithium toxicity. ECT was urgently commenced under the Mental Health Act, with a bifrontal standard stimulus approach utilised and using a MECTA Spectrum 5000Q ECT machine. Treatments were fully modified using suxamethonium and propofol with doses of 40 and 100 mg, respectively.

During planning of treatment, it was noted that the existing titration protocols guide dosing based on binary male or female gender. This posed a question for the service about what protocol should be followed in LJ's case where, although they were of female sex at birth, had been receiving testosterone GAHT for 12 months which we considered may have biological effects impacting neuromodulation treatments. In LJ's case, the threshold was 48 millicoulombs (mC) and they received the treatment dose at 72 mC with a 3 s well-modified motor seizure and 18 s electroencephalogram (EEG) seizure recorded. Following the first treatment, LJ did experience transient tachycardia to a heart rate of 108 beats per minute and was not observed on subsequent treatments. They had a normal electrocardiogram (ECG) during this time. LJ's post-ECT recovery was uneventful with no evidence of postictal confusion. Improvement in mental state was noted following the first treatment, and LJ received seven further treatments over 3 weeks with the treatment dose remaining at 72 mC. Following the fifth treatment, they were assessed to have the capacity to continue ECT under informed consent. Testosterone GAHT was continued during this time. No cognitive changes were noted across serial Montreal Cognitive Assessment testing. Following the final ECT treatment, they were discharged home with community mental health service follow-up to continue treatment with lithium as a mood stabilising agent and olanzapine antipsychotic therapy. In the months following their recovery, they continued to improve and engage with the community mental health team with plans to slowly return to work.

## 3. Literature Review

### 3.1. Method

A literature search of the Medline, Embase, and Pubmed electronic databases was performed with combinations of keywords including “electroconvulsive therapy,” “ECT,” “transgender,” “TGD,” “transexual,” “intersex,” “queer,” “gender dysphoria,” “bisexual,” “gay,” “lesbian,” “LGBTQ” (lesbian, gay, bisexual, transgender, and queer), and “gender diverse,” following systematic reviews and meta-analyses (PRISMA) guidelines. Search terms included broader gender terms to capture TGD individuals who may have been included in data under a LGBTQI + label, and the date range was from inception to August 7, 2023. References of included articles were also reviewed. The yields for these searches were as follows:

- Pubmed 57.
- Embase 24.
- Ovid MEDLINE 16.

Abstracts were screened and studies were included if they:

- included patients receiving ECT,
- included patients who were TGD, and
- included a DSM 5 or ICD 11 diagnosis.

Results were excluded if they:

- were duplicates,
- did not involve a patient sample (e.g., a letter to the editor).

The above process yielded eight studies summarised in Table 1.

### 3.2. Results

Four case reports, three case series, and one phenomenological qualitative study were identified. Of note, there were no studies including an Australian sample.

Johnstone [8] presented a phenomenological study in 1999 that explored psychological reactions of patients to ECT, including that of a TGD female to male individual. However, as the responses were qualitative and anonymised, no specific information around the TGD individual was reported.

The earliest identified case report was from Coffey and Stevens [9], who in 2016 reported the safe and effective use of ECT to treat a TGD female to male receiving GAHT who had presented with MDD. The authors noted no previous literature was identified as part of their review.

The potential interference of exogenous testosterone supplementation with anaesthetic agents used during ECT was reported by Abubucker et al. [10]. In an individual case report, these authors highlighted the case of a TGD female to male person receiving exogenous testosterone who developed bradycardia leading to asystole during ECT. They hypothesised the testosterone may have accentuated the bradycardic effect of remifentanil used during ECT as testosterone can shift the autonomic nervous system towards parasympathetic activation in females of biological sex. In another individual, case report Tran et al. [11] described the case of a TGD male to female person receiving exogenous oestrogen who developed prolonged apnoea following ECT and was found to have reduced serum pseudocholinesterase activity. These authors noted oestrogen compounds may decrease pseudocholinesterase levels leading to prolonged muscle paralysis in cases where succinylcholine is utilised.

Sauvaget et al. [12] presented a case report on an individual TGD female to male person not receiving GAHT who received treatment of their MDD with the combination of antidepressants and transcranial direct current stimulation as a further example of successful neuromodulation treatment used in this population.

Three case series were identified and yielded a combined sample of 84 individuals. However, as outlined in Table 1, Luccarelli et al. [13] and Oka et al. [14] also included members of the LGBTQ community and other gender identities without commenting specifically on the use of GAHT. Luccarelli et al. [13] found a reduction in depressive symptoms as measured by the Quick Inventory of Depressive Symptomatology in 19 patients with a mix of MDD and bipolar affective disorder. Similarly, Oka et al. [14] found a significant clinical improvement comparable to a control sample of non-LGBTQ patients receiving ECT, while Mormando et al. [15] outlined six patients with multiple psychiatric diagnoses who were refractory to pharmacologic intervention while receiving GAHT. In keeping with the two aforementioned case series, they found ECT helped improve depressive symptomatology (using Beck Depression Inventory) and observed a reduction in suicidality.

## 4. Discussion

This case report illustrates the safe and effective use of ECT in a TGD patient receiving ECT. Psychiatrists are increasingly likely to encounter patients receiving GAHT due to increased access to these medications and to the disproportionate rates of severe mental illness in the TGD population. As demonstrated above, the evidence base examining GAHT and psychiatric treatments such as ECT is sparse, with only eight studies with few participants identified in this literature review. Of these eight studies, two were case reports and identified potential for serious patient harm in the form of asystole and prolonged apnoea due to interactions between GAHT and anaesthetic medication needed for safe ECT administration. Similarly, whilst there are increasingly recognised potential biological effects of GAHT or prescribed female sex hormones on brain structure and function, the implications this has for neuromodulation techniques are not well defined in the existing literature [16]. This was relevant in the case of LJ, as current titration protocols suggest dosing based on binary male or female sex. This raises the question of which protocol should be followed and whether this may also influence individual outcomes. Without further elucidation of these interactions, psychiatrists and those TGD patients receiving ECT will remain uninformed of the potential risks, which could result in serious patient harm.

A barrier to overcome in addressing this for health services is the accurate capturing of gender data for TGD patients. For example, our local service's existing system of gender registration for ECT limited identification of previous similar cases. Similarly, a heterogeneous terminology was observed for capturing the identity of TGD individuals in the literature review and may be reflective of the wider challenges in capturing TGD health data. Other health services may consider such questions as to how to accurately capture the gender data of their own local TGD patients, which may pave the way for further crucial data on safety and efficacy of ECT treatment in this population already faced with mental health inequalities.

## Figures and Tables

**Table 1:** Summary of the literature search on the use of ECT in TGD individuals including use of GAHT.

Study	Year	Country	Design	Sample size	Sample composition	Use of gender affirming hormone therapy	Psychiatric diagnosis	Main result
Abubucker et al. [10]	2020	USA	Case report	1	Transgender female to male	Yes	BPAD	Drug interaction between remifentanil and exogenous testosterone
Tran et al. [11]	2016	USA	Case report	1	Transgender male to female	Yes	MDD	Drug interaction between exogenous oestrogen and succinylcholine
Mormando et al. [15]	2020	USA	Case series/retrospective chart review	6	Sexual identity: four males, two females	Yes, in 3 (2 estradiol and 1 testosterone)	MDD, gender dysphoria, PTSD, generalised anxiety disorder, obsessive compulsive disorder, and substance use disorder	ECT reduced depressive symptomatology and acute suicidality
Coffey and Stevens [9]	2016	USA	Case report	1	Transgender female to male	Yes	MDD	Safe and effective ECT use in a transgender female to male patient
Luccarelli et al. [13]	2021	USA	Case series	19	Patients who selected “other” for their gender: Nonbinary 9 Agender 6 Demiboy 2 Transmasculine 1 Transgender female 1 Transgender male 1 Androgynous 1 Transalienation 1 Genderqueer 1	No	MDD, BPAD, and “other”	ECT was associated with a decrease in depressive symptoms as measured by the QIDS (quick inventory of depressive symptomatology)
Sauvaget et al. [12]	2023	France	Case report	1	Transgender female to male	No	MDD	tDCS (transcranial direct current stimulation) and antidepressant combination therapy used to treat MDD in a transgender female to male patient
Oka et al. [14]	2022	USA	Case series/retrospective chart review	59	Included lesbian, gay, bisexual, and “other” (LGBQ) individuals: Female 27 Male 16 Transgender female 5 Transgender male 3 Other 5 Choose not to disclose 3 Nonbinary 0 Transgender individuals = 8	No	MDD (*n* = 41) BPAD (*n* = 15) Schizophrenia or schizoaffective disorder (*n* = 3)	Compared outcomes of LGBTQ and control sample (*n* = 441) with mood disorders receiving ECT. Found that patients experienced clinically significant improvement with ECT regardless of sexual orientation or gender identity
Johnstone et al. [8]	1999	United Kingdom	Phenomenological qualitative study	20	Transgender female to male	No	Not specified	Explored psychological reactions consumers had to ECT

## Data Availability

Supporting data can be accessed by the reference list provided. Any further details can be requested by contacting the corresponding author.
